# Alternative Mechanisms for Tn*5* Transposition

**DOI:** 10.1371/journal.pgen.1000619

**Published:** 2009-08-28

**Authors:** Asad Ahmed

**Affiliations:** Department of Biological Sciences, University of Alberta, Edmonton, Alberta, Canada; Baylor College of Medicine, United States of America

## Abstract

Bacterial transposons are known to move to new genomic sites using either a replicative or a conservative mechanism. The behavior of transposon Tn*5* is anomalous. In vitro studies indicate that it uses a conservative mechanism while in vivo results point to a replicative mechanism. To explain this anomaly, a model is presented in which the two mechanisms are not independent—as widely believed—but could represent alternate outcomes of a common transpositional pathway.

## Transposition Mechanisms in Bacteria

**Figure 1:**
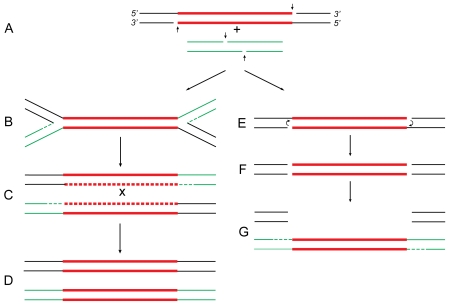
Proposed relationship of replicative and conservative transposition. Both processes start by nicking (short vertical arrows) of the transposon ends to expose the 3′-OH termini (A). At some point (see below), the target DNA is also cleaved to provide short protruding 5′-PO4 ends. In replicative transposition (left), strand-transfer takes place by joining the 3′ ends to 5′ ends of the target DNA in a concerted cleavage and joining reaction to form the “Shapiro intermediate” (B). As a result of replication of the intermediate, the donor and recipient replicons become fused to form a cointegrate (C) carrying one directly repeated copy of the transposon at each junction. Consequently, the cointegrate is an unstable structure that is resolved by *recA*-dependent generalized recombination (as in Tn*5*; A. Ahmed, unpublished results) or *tnpR*-specified site-specific recombination (as in Tn*3* [22]). The donor and recipient replicons are thereby separated, each harboring one copy of the transposon (D). If the target DNA is located within the donor replicon itself (intramolecular transposition), maturation of the Shapiro intermediate produces a replicative inversion (as shown in Figure 2) or an adjacent deletion (Figure 3). This process is highly efficient in transposons like Mu and Tn*3* [2],[9]. In conservative transposition (right), the 3′ ends engage in hairpin formation at both ends of the transposon (E) [7]. Following hairpin resolution (F), the free 3′ ends of the excised transposon are joined to 5′ ends from the target DNA (G), and the gaps are filled to complete the insertion process. The fate of the donor DNA containing a large gap (G) is not known: it could be degraded or undergo double-strand gap repair to regenerate the transposon sequence. This process is highly efficient in transposons like Tn*10* [4],[8]. In Tn*5*, hairpin formation is not efficient (i.e., is leaky), so that a small proportion of the initial 3′ nicks remains free to engage in strand-transfer. Hence, the transposon displays properties of both conservative and replicative transposition concomitantly [5],[6]. These reactions are carried out by the respective transposases, which, by oligomerization, bring the end sequences of the transposon together to form a synaptic complex. For clarity, however, the transposon is shown as a straight line. The donor DNA sequence is shown in black, transposon DNA sequence is in red, and the recipient DNA sequence is in green. Replication and gap repair are indicated by dashed lines. The crossover event that resolves the cointegrate (C) is indicated by “x.”

Transposable elements, or transposons, are discrete segments of DNA that move to many genomic sites and promote genetic rearrangements. In bacteria, they often harbor genes for antibiotic resistance that can cause serious health problems. Tn*5* is one such transposon, 5.8 kb in length, that contains a pair of inverted 1.5-kb IS*50* elements (L and R) flanking genes for kanamycin, bleomycin, and streptomycin resistance (reviewed in [1]). Bacterial transposons have been shown to use two different mechanisms, replicative and conservative (non-replicative), for their movement to new sites. The replicative mechanism (see [2],[3] and references therein), used by elements like Tn*3* and bacteriophage Mu, starts by symmetric nicking of the element to expose the 3′-OH termini (Figure 1A), which are joined to 5′-PO4 ends from the target DNA to produce a branched structure called the “Shapiro intermediate” (Figure 1B). Replication of this structure from forks created at both ends of the transposon results in the formation of two copies of the element (Figure 1C), one of which ultimately appears at the target DNA site while the other remains at the original donor DNA site (Figure 1D). This scheme explains the formation of all genetic rearrangements known to be associated with these elements. The conservative mechanism, also called the “cut-and-paste” mechanism, is used by elements like Tn*10* (reviewed in [4]). The element is excised cleanly by double-strand cleavages from the donor DNA (Figures 1E and 1F) and inserted, with limited repair, between a pair of staggered nicks at the target DNA (Figure 1G). This mechanism in turn accounts for all of the specific rearrangements observed with Tn*10*. The evidence for Tn*5* is, however, mixed. Biochemical evidence indicates a mechanism similar to Tn*10* [5], while genetic evidence indicates strong similarities to Tn*3* and Mu [6]. Here I show that the anomalous behavior of Tn*5* indicates that the replicative and conservative mechanisms may not be independent pathways, as believed previously, but alternate outcomes of a common pathway.

## Definitions

**Figure 2:**
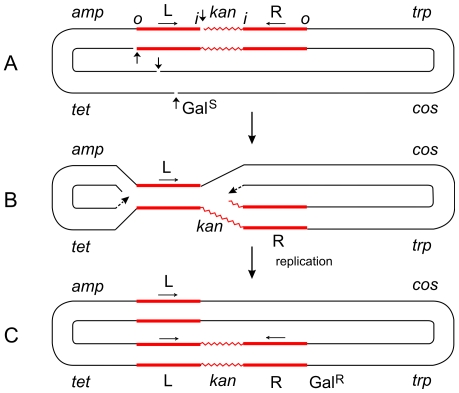
Steps in the formation of the Tn*5*-promoted replicative inversion *621*. The parent plasmid p4.1 (A) carried one copy of Tn*5* (consisting of two inverted IS*50* elements, L and R, flanking the *kan* gene), the *trp*, *galTK*, *tet*, and *amp* genes, and the *cos* site of lambda [6]. The *galTK* genes confer galactose-sensitivity (Gal^S^) on the host cell, and selection for galactose-resistance (Gal^R^) requires the disruption of this region. In inversion *621* (C), only one IS*50* element (L) was left at the original location, a complete copy of Tn*5* was found inserted in the *gal* region, and the *trp-cos* plasmid segment between the two had been inverted. This type III inversion (like several type I and II inversions also described in [6]) is fully consistent with the replicative mechanism as depicted in (B). It cannot be explained by the conservative mechanism. Small vertical arrows indicate location of nicks at the ends of IS*50*L and the target (*galTK*) DNA sequence. Horizontal arrows indicate inverted orientation of the two IS*50* elements. The letters “*o*” and “*I*” refer to the outside and inside ends of the transposon, respectively. Figures are not drawn to scale. A deletion-inversion arising from p4.1 would be expected to have the following structure: the central *kan* region of Tn*5* would be deleted, IS*50*L would join the *gal* region, the *trp-cos* segment would be inverted, and IS*50*R would also be inverted (to produce a direct repeat of IS*50*L) and join the target site in the *gal* region. This event would be consistent with the conservative mechanism, but it was never recovered from Tn*5* in vivo. In contrast, the behavior of Tn*10* was just the opposite of Tn*5*. p6A.1, a plasmid that is similar in structure to p4.1—except that it harbors Tn*10* in place of Tn*5*—produced only deletion-inversions and no replicative inversions [6]. The two inside ends of Tn*10* would be cleaved by double-strand breaks (as a result of hairpin formation and resolution), and the free 3′ ends would attack and join 5′ ends of the target sequence from the opposite strand. Such an event would produce deletion-inversions of the prescribed structure.

**Figure 3:**
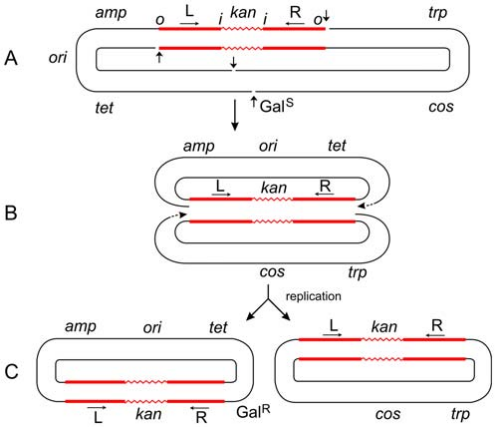
Steps in the formation of Tn*5*-promoted adjacent deletions. The plasmid p4.1 (A) carrying Tn*5* was used for the selection of deletions conferring galactose-resistance (Gal^R^). Using the replicative mechanism, Tn*5* would be nicked at its termini to produce 3′ ends that would attack the target DNA sequence and join the 5′ ends from the same strand. This would result in the formation of a Shapiro intermediate containing replication forks at both ends of the transposon (B). After replication is completed, two deletion circles would be formed (C), only one of which would carry the origin of replication (*ori*) and survive. Thus a series of overlapping deletions starting from a fixed site at the right transposon terminus and extending to various sites in the *gal* region and beyond can be selected positively as Gal^R^ colonies. This has been the basis for the development of vectors for DNA sequencing [23]. The Shapiro intermediate can also be formed at individual IS elements (for instance, IS*50*L) to produce deletions extending from an inside end of the transposon. However, the majority (95%) of deletions in Tn*5* start from the outside end. If Tn*5* transposed solely by the conservative mechanism, both outside ends of the transposon would be cleaved by double-strand breaks; so, no viable deletion products would be formed after strand-transfer since the plasmid backbone would have been cut at the other end too. That such deletions are actually recovered in large numbers suggests that Tn*5* can also utilize the replicative mechanism for its transposition. The plasmid, p6A.1, which carries Tn*10* instead of Tn*5*, behaves in a different manner. It produced deletions solely from an inside end, and none from the outside end [6]. This behavior is to be expected since Tn*10* uses the conservative mechanism, and double-strand cuts made at the outside ends would generate inviable deletion products. On the other hand, double-strand cuts made at the two inside ends of Tn*10* would generate viable products. The 3′ ends from the inside ends would attack the target sequence and join 5′ ends from the same strand to produce two deletion circles, only one of which would carry *ori* and survive. This is actually found to be the case. (If the 3′ ends from the inside ends joined the 5′ ends from the opposite strand, the result would be a deletion-inversion as described in the legend to Figure 2.) Hence, the difference between the formation of transposon-promoted deletions and inversions is very narrow and depends on the topology of strand attacks: same-strand attacks produce two deletion circles; opposite-strand attacks produce an inversion circle [24].

For clarity, the intermediates and genetic rearrangements associated with bacterial transposition are defined first. Composite transposons like Tn*5* and Tn*10* typically consist of two copies of IS (insertion sequence) elements flanking a central region containing various antibiotic-resistance genes. (In both Tn*5* and Tn*10*, the IS elements are present as inverted repeats.) The replicon that harbors the transposon is referred to as the “donor” replicon, and the one that receives it is referred to as the “recipient” replicon. A transposon inserted in the “target” DNA site of the recipient without any associated rearrangement is referred to as a “simple insert.” Still, a simple insert carries a short (usually five or nine base pairs) duplication of the target sequence at both ends. This is due to the formation of staggered nicks at the target site during the insertion process. Simple inserts can arise through a “cut-and-paste” process (which does not involve replication), resolution of cointegrates (which involves replication), or, possibly, the breakdown of the “Shapiro intermediate.” During replicative transposition, the Shapiro intermediate is replicated to form a composite structure called the “cointegrate” in which the donor and recipient replicons are fused together with one copy of the transposon present at each junction. Since the two copies of the transposon are arranged as direct repeats, the cointegrate is an unstable intermediate which is “resolved” into the donor and recipient replicons—each harboring one copy of the transposon. Resolution of the cointegrate is carried out by recombination (site-specific or generalized) between the two directly repeated copies of the transposable element. Transposons can also carry out “intramolecular transposition” (i.e., transposition at other DNA sites within the same replicon) to produce inversions or adjacent deletions. In “replicative inversions,” a new copy of the transposon appears at the target DNA site, and the DNA segment between the original and the new copy of the transposon is inverted. The other kind of inversion is “deletion-inversion,” which involves both a specific deletion of transposon DNA and an inversion. The central region of the transposon is deleted, one IS element is joined to the target, the DNA segment between the target and the second IS element is inverted, and the second IS element is connected back to the target DNA site. (Details of the structure and formation of these inversions are given in the legend to Figure 2.) Transposon-promoted “adjacent deletions” start precisely at the transposon termini and extend outwards into adjoining DNA. They can start either from the “outside” ends or the “inside” ends of the transposon. Although the final structures of inversions and deletions show profound differences, they originate in a fine difference at the crucial strand-transfer step (see legend to Figure 3). Among these rearrangements, cointegrates and replicative inversions are products unique to replicative transposition, while deletion-inversions seem unique to conservative transposition.

## Tn*5* Transposition

Extensive studies by Kleckner and colleagues have shown that conservative transposition of Tn*10* takes place in several steps [7]. These steps are: first-strand nicking to expose the 3′ ends of the transposon (Figure 1A), hairpin formation by the 3′ ends to cause second-strand nicking (Figure 1E), hairpin resolution to free the 3′ ends (Figure 1F), strand-transfer to join the free 3′ ends to 5′ ends from the target DNA (Figure 1G) and, finally, gap repair to complete the insertion process. The transposon is thus excised free from the donor by double-strand cuts and inserted within the target DNA without extensive replication. Nearly all steps in this process have been reproduced in vitro [7],[8]. This mechanism provides satisfactory explanation for various Tn*10*-specific rearrangements, viz., simple inserts, adjacent deletions, and deletion-inversions. More importantly, it also accounts for the absence of cointegrates and replicative inversions among Tn*10*-promoted rearrangements.

Subsequent biochemical studies by Reznikoff and co-workers have shown that Tn*5* uses a mechanism essentially similar to Tn*10*. Hence, they concluded that Tn*5* transposition is also conservative. The details of this process have been reviewed recently [5].

## The Tn*5* Paradox

A puzzling feature of Tn*5* that has remained elusive, however, is that the genetic rearrangements promoted by Tn*5* are strikingly different from those promoted by Tn*10*. Not only that, they are completely identical to those promoted by Tn*3* and Mu—elements that use a replicative mechanism [6]. Biochemical studies by Mizuuchi and collaborators confirmed that replicative transposition of Mu takes place by first-strand nicking to expose the 3′ ends of the element, strand-transfer to join the 3′ ends to 5′ ends from the cleaved target to form the Shapiro intermediate, and replication from one or both ends of the transposon to produce two copies of the element (Figures 1A–1D, reviewed in [9]). If the donor and target DNAs lie on separate replicons, a fusion structure (the cointegrate) is formed; if they lie on the same replicon, the result is either a replicative inversion or an adjacent deletion depending on target orientation [2]. Most of these steps have also been reproduced in vitro [10]–[12]. This mechanism explains the formation of cointegrates, simple inserts, adjacent deletions, and replicative inversions normally observed with Mu and Tn*3*. The point that needs to be stressed here is that, in both cointegrates and replicative inversions, a duplicate copy of the transposon is recovered at the target DNA site. This is strong genetic evidence to show that the transposon does undergo replication during its movement.

In a comparative study of transposons Tn*5* and Tn*10*, all of the genetic rearrangements known to be associated with replicative transposition (cointegrates, simple inserts, adjacent deletions, and replicative inversions) were also recovered from Tn*5* [6]. In contrast, Tn*10* did not produce cointegrates and replicative inversions, but produced deletion-inversions as expected for the conservative mechanism [4]. As an example of Tn*5*-promoted inversions, consider the formation of the type III inversion *621* described in [6]. Starting with a single copy of Tn*5* on the parent plasmid, the inversion retains only one IS*50* element at the original site, a new copy of the entire Tn*5* transposon appears at the target site, and the plasmid segment between the two is inverted. As shown in Figure 2, a single replicative event is sufficient to explain its formation. On the other hand, this rearrangement cannot be explained as a single event by the conservative mechanism or without making special assumptions.

Another important difference lies in the nature of adjacent deletions promoted by the two transposons [6]. Deletions promoted by Tn*5* start from both outside and inside ends of the transposon and extend into adjacent DNA. (However, the outside end is strongly preferred since 95% of the deletions start there, while only 5% start from an inside end.) In contrast, all (100%) of the Tn*10*-promoted deletions start from an inside end of the transposon. As shown in Figure 3, deletions from the outside (and also inside) end of Tn*5* can be explained by the appearance of nicks and formation of the Shapiro intermediate. On the other hand, if both strands of Tn*5* were cut (as required by the conservative mechanism), attacks from the 3′ ends of the excised transposon on the target DNA would generate inviable products, since the plasmid backbone would have also been cleaved at the other end. No such constraints apply when double-strand cuts are made at the inside ends of the transposon. Hence, Tn*10* produces adjacent deletions arising only from an inside end of the transposon. The production of adjacent deletions that originate mainly from the outside ends of Tn*5* therefore suggests that they arise by a replicative, rather than a conservative, mechanism.

Furthermore, we have evidence (L. Podemski and A. Ahmed, unpublished data) that purified monomeric plasmids carrying Tn*5* produce genuine cointegrates with the F-plasmid pOX38 while those containing Tn*10* do not; dimeric plasmids from both produce cointegrate-like structures. The rates of formation [13] of simple inserts and cointegrates, respectively, by Tn*5* were 1.1×10^−7^ and 0.1×10^−7^/donor cell per division from monomeric donors (increasing to 1.9×10^−7^ and 0.7×10^−7^ from dimeric donors). Thus, 92% of the Tn*5*-promoted events are simple inserts, and only 8% are cointegrates that can be missed easily [14]. In any case, both of these rearrangements (replicative inversions and true cointegrates) are characteristically associated with replicative transposition. It may be pointed out here that the inversions generated in vitro by purified Tn*5* transposase [15] exhibit a structure similar to deletion-inversions reported from Tn*10* [4], which is clearly different from inversions produced by Tn*5* in vivo and discussed here [6]. It is also hard to reconcile these in vivo observations with the view, based on in vitro studies, that transposon Tn*5* is released free from the donor DNA by double-strand cleavages at both ends before capturing the target DNA (see Figure 3 in [5]), as is known to be the case in Tn*10* [16]. This shows that the results of in vitro studies should be extrapolated to biological phenomena with care. For instance, the conservative model for Tn*10* was developed by extensive analysis of in vivo results, and confirmed later by in vitro studies [4],[8]. To sum up, biochemical studies indicate that Tn*5* transposition is conservative while genetic studies suggest that a replicative pathway is also utilized. How could it be that Tn*5* exhibits properties of both conservative and replicative transposition concurrently?

## The Explanation

The paradox can be resolved by taking a closer look at the early steps in the transposition process (Figure 1). Both replicative and conservative processes are initiated by nicking of the transposon to expose its 3′ termini (Figure 1A) [17]. In replicative transposition, these 3′ nicks are joined, in a concerted cleavage and joining reaction [9],[11], to the 5′ ends generated from staggered nicks at the target DNA to form the Shapiro intermediate (Figure 1B). The replication fork-like structures at both ends of the transposon allow replication (Figure 1C) to proceed inwards to form two copies of the element, one of which ultimately appears at the target DNA site while the other remains at the donor DNA site (Figure 1D). In conservative transposition, the 3′ ends do not participate in strand-transfer immediately, but form hairpins at the termini that lead to second-strand nicking at both ends of the transposon (Figure 1E). Following hairpin resolution (Figure 1F), the excised transposon carries out strand-transfer from its free 3′ ends to join 5′ ends from the target DNA (Figure 1G). Since both strands of the element are inserted in the target DNA site, there is no need for further replication except for limited gap repair. Thus, the choice between replicative and conservative transposition boils down to whether the initial 3′ nicks engage in strand-transfer before, or after, the formation and resolution of hairpins. If strand-transfer occurs before hairpin formation, the result is replicative transposition; if it occurs after hairpin resolution, the result is conservative transposition. The choice between the two alternatives would depend on the nature and efficiency of the particular transposase. If a transposase carries out strand-transfer with high efficiency, the outcome is replicative as seen in Tn*3* and Mu. On the other hand, if the transposase is more efficient in hairpin formation, the outcome is conservative as seen in Tn*10*. If hairpin formation is less efficient (i.e., is “leaky”), the majority of the 3′ nicks would still participate in hairpin formation, but some would remain free to undergo strand-transfer. This seems to be the case in Tn*5*. As a result, Tn*5* displays features of both replicative and conservative transposition concurrently.

A clear prediction of this proposal is that, in Tn*5*, target capture and strand-transfer should also occur before hairpin formation. In other words, a target DNA molecule would need to be assimilated by the Tn*5* synaptic complex before release of the donor backbone. Checking this prediction would require a re-examination of the cocrystal structure of Tn*5* transposase complexed with DNA (reviewed in [5]). Also, it should be possible to isolate mutants of Tn*5* that would shift the balance of conservative/replicative transposition in either direction. In fact, Tavakoli and Derbyshire have reported several mutants of IS*903* (affecting a region close to the catalytic residues of the transposase) that increase the frequency of replicative transposition in relation to simple insertions [18]. These authors suggested that a delay in cleavage of the 5′-flanking DNA may increase the half-life of the 3′-nicked intermediate and consequently enhance cointegrate formation. To explain this observation, they proposed a scheme essentially similar to that presented in Figure 1. May and Craig have also reported that a single point mutation in the Tn*7*-coded TnsA protein can switch the mode of transposition from conservative to replicative [19]. Even the MuA transposase, which normally carries out Mu DNA replication through repeated cycles of replicative transposition, has been shown to catalyze the processing of model DNA hairpin substrates into products that are competent for strand-transfer [20]. Although the full implication of this finding is not yet clear, it should be borne in mind that Mu DNA transposition from an infecting phage into the host chromosome occurs by a mechanism that is conservative, while the subsequent transpositional events are replicative [21]. It is therefore conceivable that Mu transposition could also follow the scheme outlined in Figure 1. These findings underscore the mechanistic similarities of various transposases, and the ability to switch between alternate modes of transposition should confer evolutionary advantage for the dissemination of these transposons. Hence replicative and conservative mechanisms should not be viewed as independent pathways, but only as a reflection of the relative efficiencies of two transposase-mediated processes occurring at the 3′-OH ends: strand-transfer and hairpin formation.
